# La producción pública de antivenenos en la Región de las Américas como factor clave en su accesibilidad

**DOI:** 10.26633/RPSP.2017.109

**Published:** 2017-07-20

**Authors:** Guillermo Temprano, Patricia Aprea, José Christian Dokmetjian

**Affiliations:** 1 Instituto Nacional de Producción de Biológicos Administración Nacional de Laboratorios e Institutos de Salud – ANLIS “Dr. Carlos G. Malbrán” Buenos Aires Argentina Instituto Nacional de Producción de Biológicos, Administración Nacional de Laboratorios e Institutos de Salud – ANLIS “Dr. Carlos G. Malbrán”, Buenos Aires, Argentina.; 2 Dirección de Evaluación y Control de Biológicos y Radiofármacos Instituto Nacional de Medicamentos, Administración Nacional de Medicamentos, Alimentos y Tecnología Médica – ANMAT Buenos Aires Argentina Dirección de Evaluación y Control de Biológicos y Radiofármacos, Instituto Nacional de Medicamentos, Administración Nacional de Medicamentos, Alimentos y Tecnología Médica – ANMAT, Buenos Aires, Argentina.

**Keywords:** Antivenenos, ponzoñas, producción de medicamentos sin interés comercial, Américas, Antivenins, venoms, orphan drug production, Americas, Antivenenos, peçonhas, las Américas, produção de droga sem interesse comercial, Américas

## Abstract

Los accidentes causados por animales ponzoñosos afectan vastas zonas de América Latina, Sur de Asia, Sudeste Asiático, África subsahariana y Oceanía y representan un serio problema para la salud pública mundial. A partir de un análisis del panorama actual en materia de producción global de los antivenenos ofídicos y aracnídicos, se concluye que son productos semi-huérfanos. Esta situación plantea un escenario favorable para fortalecer su producción por parte de los laboratorios públicos. Los gobiernos deberán tomar una decisión política al respecto en aras de la equidad en la salud de la población. En la Región de las Américas, estas acciones podrían enmarcarse en un programa liderado por la Organización Panamericana de la Salud, para garantizar la disponibilidad de estos productos biológicos en centros asistenciales estratégicamente localizados. Se han identificado 12 establecimientos públicos productores de antivenenos en la Región entre los cuales los de Brasil y México son los mayores productores públicos. La gestión de estos laboratorios debe ser la propia de una organización industrial productora de bienes tangibles que no soslaye la planificación estratégica. Las autoridades regulatorias nacionales deberían ayudar a los laboratorios públicos que los producen prestándoles asesoramiento y consultoría sin perder la imparcialidad ni el rigor necesarios en la evaluación de sus sistemas de gestión de la calidad. Las nuevas tecnologías superiores de la producción a partir de plasma hiperinmune de mamíferos se encuentran en fase experimental. No se ha encontrado en la bibliografía información sobre su incorporación en las líneas de producción.

## Abstract (en)

Injuries caused by venomous animals affect vast areas of Latin America, Southern Asia, Southeast Asia, sub-Saharan Africa, and Oceania, and pose a serious problem for global public health. Based on an analysis of the current panorama of global production of ophidian and arachnid antivenoms, it is concluded that they are semi-orphaned products. This is a favorable scenario in which to strengthen public laboratory production. Governments should make a political decision in this regard in the interest of equity in population health. In the Region of the Americas, these actions could be part of a program led by the Pan American Health Organization to ensure the availability of these biologicals in strategically located health centers. Twelve public facilities producing antivenoms have been identified in the Region, including Brazil and Mexico, which are the biggest public producers. These laboratories should be managed like industrial operations that produce tangible goods without ignoring strategic planning. National regulatory authorities should help the public laboratories that produce them by providing necessary technical assistance and consultancy without any loss of impartiality or rigor in the evaluation of their quality management systems. New superior production technologies using hyperimmune mammalian plasma are in the experimental phase; no information on its production has been found in the literature.

## RESUMO

Os acidentes causados por animais peçonhentos se distribuem por vastas áreas da América Latina, sul da Ásia, Sudeste Asiático, África subsaariana e Oceania, representando um sério problema à saúde pública mundial. A partir de uma análise do panorama atual da produção mundial de soros antiofídicos e antiaracnídeos se concluiu que são produtos semiórfãos. Tal situação cria um cenário favorável para estimular a produção destes produtos por laboratórios públicos. Os governos devem tomar uma decisão política neste sentido para assegurar a equidade em saúde da população. Na Região das Américas, as ações poderiam englobar um programa encabeçado pela Organização Pan-Americana da Saúde para garantir a disponibilidade dos produtos biológicos em centros de atenção estrategicamente localizados. Existem 12 instituições públicas que produzem soros antivenenos na Região, sendo que os maiores produtores estão no Brasil e no México. Estes laboratórios devem ser geridos como uma indústria produtora de bens tangíveis sujeita a um planejamento estratégico. Os órgãos reguladores nacionais devem auxiliar os laboratórios públicos prestando assessoria e consultoria com a imparcialidade e o rigor necessários para avaliar a gestão da qualidade. Novas tecnologias avançadas de produção de soro baseadas em plasma hiperimune de mamíferos estão em fase experimental. Não há informação na literatura sobre a inclusão destas tecnologias nas linhas de produção.

Los accidentes causados por animales ponzoñosos afectan vastas zonas de América Latina, Sur de Asia, Sudeste Asiático, África subsahariana y Oceanía y representan un serio problema para la salud pública global (1–4). Respecto a los ofidios, según la Global Snakebite Initiative*,* cada año en el mundo se producen al menos 421 000 envenenamientos y 20 000 muertes, unas estimaciones que pueden incluso alcanzar los 1 841 000 y las 94 000, respectivamente. Considerando que el envenenamiento ocurre casi en una de cuatro mordeduras, puede inferirse que anualmente se producen entre 1,2 y 5,5 millones de mordeduras (2). Además de los casos de óbito, deben considerarse los de las discapacidades físicas permanentes que causan.

A pesar de ser un problema de salud de alto impacto global, actualmente no se encuentra clasificado como enfermedad tropical desatendida (neglected tropical disease) en la lista oficial de la Organización Mundial de la Salud (OMS) (5). Un estudio sobre su incidencia en África Occidental indica que su frecuencia es mayor que la de otras enfermedades incluidas en dicha lista y por este motivo está subestimada (6). Sin embargo, en un taller de expertos que tuvo lugar en el Reino Unido en 2015 (7) se concluyó que, si bien las mordeduras por serpientes se han categorizado históricamente como accidentes o injurias, constituyen una enfermedad tropical desatendida.

Por otra parte, los antivenenos para el tratamiento de accidentes (también denominados sueros terapéuticos) sí están incluidos en la lista de medicamentos esenciales de la OMS (8). Su ingrediente farmacéutico activo está compuesto por inmunoglobulinas o fragmentos de ellas obtenidos a partir del suero de animales hiperinmunizados con venenos. Actualmente constituyen la principal herramienta eficaz y segura para disminuir la morbimortalidad causada por accidentes por animales venenosos, sin menoscabar las demás acciones terapéuticas que indique el médico. Su calidad y disponibilidad en el lugar y en el momento en que se necesite por indicación médica son factores determinantes de la supervivencia del paciente accidentado. Para mejorar su disponibilidad, se han sugerido acciones a escala global como la preparación de *pooles* representativos y validados de venenos, el fortalecimiento de la manufactura y el control de calidad, la generación de antivenenos de uso internacional, políticas públicas que den un marco adecuado de referencia, y otras medidas de mitigación (9).

En septiembre de 2015, la ONG Médicos Sin Fronteras anunció que el último lote del antiveneno multivalente producido solamente por una compañía farmacéutica internacional expiraba en junio de 2016 (10,11); su manufactura se paralizó en 2014. Otras dos compañías farmacéuticas líderes están adoptando decisiones similares. Por lo expuesto, los antivenenos se han convertido en productos semi-huérfanos**,** entendidos como productos huérfanos en la práctica, no como medicamentos indicados para el tratamiento de enfermedades raras.

Los objetivos de este trabajo son: señalar la necesidad de fortalecer la capacidad de producción de antivenenos de los laboratorios públicos; indicar la conveniencia de la adopción por parte de la Organización Panamericana de la Salud (OPS) de un programa de alcance regional para afianzar la producción de antivenenos y su distribución; identificar los laboratorios públicos productores de antivenenos en la Región de las Américas y comparar sus escalas de producción; proponer acciones en gestión institucional de los laboratorios públicos y en el papel de las autoridades regulatorias nacionales, y destacar la importancia de las nuevas tecnologías en el desarrollo de antivenenos.

## PRODUCCIÓN PÚBLICA EN LAS AMÉRICAS

El Centro de Produção e Pesquisa de Imunobiológicos del estado de Paraná en Brasil está desarrollando un proyecto de edificación de una nueva planta de producción, para observar el cumplimiento de las normas de Buenas Prácticas de Fabricación. Su producción anual es fluctuante. Los tres laboratorios productores de antivenenos restantes de Brasil (el Instituto Butantan del estado de Sâo Paulo, la Fundação Ezequiel Dias, de Minas Gerais y el Instituto Vital Brazil del de Río de Janeiro) presentan escalas de producción de orden similar. El cuadro 1 muestra que Brasil y México son los mayores productores públicos de antivenenos en la Región.

En Argentina, el mayor productor en cantidad y variedad de antivenenos es el Instituto Nacional de Producción de Biológicos, que forma parte de la Administración Nacional de Laboratorios e Institutos de Salud “Dr. Carlos G. Malbrán”. Esta Administración es miembro de la International Association of National Public Health Institutes (IANPHI). Por su parte, el Instituto Biológico “Dr. Tomás Perón”, de la provincia de Buenos Aires, suple las necesidades de dicha jurisdicción acotado a su cartera de antivenenos.

En Bolivia, Perú y Colombia los productores también son los respectivos institutos nacionales de salud, todos ellos miembros asimismo de la IANPHI. En México, el laboratorio de producción pública de antivenenos (Birmex) es una sociedad anónima de capital variable, con mayoría accionarial del Estado.

El único laboratorio productor en Venezuela (Biotecfar) es una empresa de capital accionarial perteneciente a la Universidad Central. El Instituto Clodomiro Picado se encuentra bajo jurisdicción de la Universidad de Costa Rica. Su volumen de producción permite abastecer no sólo las demandas del país, sino que exporta a otros países de Centroamérica. Cabe destacar su capacidad para producir antivenenos de serpientes del África subsahariana. Los restantes países de la Región no tienen producción pública de antivenenos.

La situación en materia de producción de antivenenos se contrapone con la de las vacunas en la Región, donde unas pocas compañías farmacéuticas líderes tienen un marcado interés (12). Atendiendo a su condición de productos semi-huérfanos, se impone que las autoridades de salud de los países acometan acciones dirigidas a fortalecer las capacidades de producción de los laboratorios públicos. A escala regional, estas acciones deberían enmarcarse en un programa gestionado por la OPS dirigido a garantizar la disponibilidad de estos productos biológicos en centros asistenciales estratégicamente localizados.

## GESTIÓN INSTITUCIONAL

Como muestra el cuadro 1, en Argentina, Bolivia, Colombia y Perú los laboratorios de producción pública de antivenenos están enmarcados en los institutos nacionales de salud. Esto significa que se rigen por la legislación correspondiente de la Administración Pública Nacional. En Brasil, son laboratorios dependientes de los estados federados. Si bien se observaron avances en la gestión pública en la Región, como ejemplifican los mecanismos de compra y evaluación de proveedores, es importante subrayar que, para cumplir con su responsabilidad primaria, la gestión de estos laboratorios debe ser la propia de una organización industrial productora de bienes tangibles. Según Henry Mintzberg, la configuración de estas organizaciones se corresponde con la de una burocracia mecanizada o maquinal (13). En este tipo de organizaciones es clave la tecnoestructura, que está integrada por aquellas personas (analistas) situadas fuera de la línea jerárquica y se encargan de estandarizar o normalizar los procesos de trabajo. Están fuera de la corriente de trabajo operacional; pueden diseñarla, planearla, cambiarla o entrenar al personal, pero no lo hacen ellas mismas, lo que facilita el cumplimiento integral de las normas de Buenas Prácticas de Fabricación, de Buenas Prácticas de Laboratorio, de Buenas Prácticas Clínicas y las normas ISO 9001:2015 (14) e ISO/IEC 17.025:2005 (15), todas ellas aplicables, ya sea dentro del ámbito regulado o voluntario, a un laboratorio de estas características.

**CUADRO I.** Producción pública de antivenenos ofídicos y aracnídicos en la Región de las Américas

País	Establecimiento Productor	Géneros involucrados	Unidades producidas por año
Argentina	Instituto Nacional de Producción de Biológicos Administración Nacional de Laboratorios e Institutos de Salud ANLIS “Dr. Carlos MALBRÁN”	Bothrops	22 000
		Crotalus	
		Micrurus	
		Loxosceles	
		Latrodectus	
		Tityus	
	Instituto Biológico “Dr. Tomás PERÓN”	Bothrops	3 000
		Loxosceles	
		Latrodectus	
Bolivia	Instituto Nacional de Laboratorios de Salud - INLASA	Bothrops	5 000
		Crotalus	
		Lachesis	
Brasil	Instituto Butantán	Bothrops	226 000
		Crotalus	
		Micrurus	
		Lachesis	
		Loxosceles	
		Phoneutria	
		Tityus	
		Lonomia	
	Instituto Vital Brazil	Bothrops	250 000
		Crotalus	
		Lachesis	
		Tityus	
	Fundação Ezequiel Dias	Bothrops	207 000
		Crotalus	
		Micrurus	
		Lachesis	
		Tityus	
	Centro de Produção e Pesquisa de Imunobiológicos	Bothrops	Fluctuante
		Loxosceles	
Colombia	Instituto Nacional de Salud	Bothrops	11 000
		Crotalus	
		Lachesis	
Costa Rica	Instituto Clodomiro Picado	Bothrops	100 000
		Crotalus	
		Micrurus	
		Lachesis	
		*Vipéridos y*	
		*elápidos*	
		*subsaharianos*	
México	Birmex	Bothrops	495 000
		Crotalus	
		Centruroides	
Perú	Instituto Nacional de Salud	Bothrops	17 600
		Crotalus	
		Lachesis	
		Loxosceles	
Venezuela	Biotecfar	Bothrops	72 500
		Crotalus	
		Porthidium	
		Tityus	

***Fuente:*** Taller de antivenenos regional FEMCIDI-OEA. San Pablo, Brasil, 2014 y consultas a establecimientos productores.

Para que los laboratorios puedan cumplir con su misión y alcanzar su visión, la gestión institucional no puede soslayar la planificación estratégica, a partir de la cual se delinearán estrategias, metas y planes. En el ámbito público, un método particularmente interesante es el de la Planificación Estratégica Situacional (PES): “Es un cuerpo teórico-metodológico-práctico muy sólido, sistemático y riguroso” […] “El PES tiene en común con la rama buena de la planificación estratégica corporativa su consideración de varios actores en juego de conflicto y cooperación. Pero se diferencia de ella en que los actores son partidos políticos, gobernantes o dirigentes de organizaciones públicas, empresariales y sindicales. El PES es un método y una teoría de Planificación Estratégica Pública, la más nueva de las ramas de la planificación estratégica” (16).

Todo ello supone un compromiso por parte de los gobiernos de promover la producción pública de estos medicamentos biológicos. A su vez, para generar confianza por parte de los gobiernos en el abastecimiento al sistema público de salud, las direcciones o gerencias de los laboratorios productores deberán crear las condiciones idóneas para conseguir una producción predecible, sostenida, a escala industrial y atendiendo a la totalidad o a una fracción significativa preestablecida de la demanda del sistema público de salud. También es conveniente que la gestión se enfoque conforme a los siete principios de gestión de la calidad enunciados en la norma ISO 9000:2015: enfoque al cliente, liderazgo, compromiso de las personas, enfoque basado en procesos, mejora, toma de decisiones basada en las evidencias, y gestión de las relaciones (17).

## PAPEL DE LAS AUTORIDADES REGULATORIAS EN LA REGIÓN

Varios laboratorios productores de la Región tienen dificultades para cumplir integralmente las normas de Buenas Prácticas de Fabricación vigentes en sus países. Para ello, además de contar con áreas con *lay-out* e instalaciones conformes con tales normativas, deberán modificar sus prácticas de trabajo. En algunos casos será conveniente acometer una reforma en la estructura organizativa: “Para reformar una organización se deben cambiar las prácticas de trabajo por la vía activa, o sea, la mudanza de la cultura institucional. Pero si la mudanza está trabada pasivamente por las formas organizativas, es necesario liberar las restricciones formales”… “Cuando las formas organizativas son muy viejas y consolidadas, la carga pasiva puede ser muy fuerte” (18)*.*

Para hacer frente a este problema, las autoridades reguladoras de cada país deberían ayudar a los laboratorios públicos productores de antivenenos prestándoles asesoramiento y consultoría por medio de los cuales, sin perder la imparcialidad ni el rigor necesarios al inspeccionar y evaluar los laboratorios y sus sistemas de gestión de la calidad, estas organizaciones puedan estar a la altura de todo laboratorio productor industrial de medicamentos. Un ejemplo de este vínculo es un proyecto de innovación suscrito entre el Instituto Nacional de Producción de Biológicos, perteneciente a ANLIS “Dr. Carlos Malbrán” y la autoridad reguladora de Argentina, la Administración Nacional de Medicamentos, Alimentos y Tecnología Médica (ANMAT).

## DESARROLLO DE NUEVAS TECNOLOGÍAS

En febrero de 2014 se celebró en el Instituto Butantan, en Sâo Paulo, Brasil, el Taller Producción de antivenenos en laboratorios públicos de América Latina: situación actual, aspectos metrológicos y necesidades de cooperación regional, en el contexto de un proyecto FEMCIDI-OEA. En dicho taller participaron diez de los doce laboratorios productores incluidos en el cuadro 1. De las ponencias de dicho evento se concluye que todos los laboratorios participantes emplean como material de partida plasma hiperinmune de mamíferos y que el fraccionamiento del plasma se lleva a cabo mediante precipitación (o doble precipitación) con sulfato de amonio, ácido caprílico o ambos.

A pesar de los enormes avances en venómica desde fines del siglo XX, aún no se ha desarrollado una alternativa innovadora a los antivenenos derivados de plasma de animales hiperinmunes. Los antivenenos disponibles neutralizan indiscriminadamente proteínas tóxicas y no tóxicas de venenos. Para obtener un producto superior, varios grupos de científicos están trabajando en la materia con estrategias que, generalizando, se orientan en dos sentidos: a) mejorar el inmunógeno y b) optimizar los procesos o el cambio tecnológico en la obtención del antiveneno. La primera incluye el análisis estructural de los venenos (perfiles proteómicos y de actividad biológica). Es posible identificar las proteínas responsables de la actividad biológica del veneno de una determinada especie y en un futuro próximo inmunizar al animal productor del plasma (material de partida del antiveneno) con un antígeno compuesto por las proteínas seleccionadas (19), así como obtener estas proteínas mediante transcriptómica (20) u organismos genéticamente modificados (21,22). En cuanto a la optimización de los procesos, ya está puesto a punto en líneas de producción el método de precipitación única con ácido caprílico, que tiene como ventaja su mayor rendimiento respecto al de doble precipitación con sulfato de amonio (23).

En lo relativo al desarrollo de nuevas estrategias o tecnologías, que se encuentran en fase experimental, cabe mencionar las siguientes:

- Se ha aislado una proteína, denominada DM43, del suero de zarigüeya (comadreja, *Didelphis marsupialis*) que inhibe parcialmente la actividad hemorrágica de metaloproteinasas de *Bohtrops jararacá* (24);
- se ha preparado un aerosol nasal de neostigmina que es eficaz en modelos de ratones y humanos para revertir la parálisis en el tratamiento temprano de envenenamientos neurotóxicos (25, 26). Con esta estrategia se pretende hallar un antídoto universal para todo tipo de toxinas neurotóxicas;
- se estudia aplicar la denominada Tecnología IgY para obtener antivenenos (27, 28). Las inmunoglobulinas IgY obtenidas a partir de yema de huevo presentan la ventaja, respecto a las inmunoglobulinas de mamíferos, de no fijar el complemento ni factores reumatoideos. Esta tecnología tiene un alto rendimiento con factibilidad de bioterio con aves libres de patógenos específicos.

Por último, es preciso fortalecer mecanismos de cooperación entre los laboratorios, tanto en los aspectos tecnológicos como en los de gestión y, en este sentido, debe destacarse el proyecto en curso que se enmarca en el Fondo Especial Multilateral del Consejo Interamericano para el Desarrollo Integral patrocinado por la Organización de Estados Americanos (FEMCIDI). Este Fondo se creó en 1997 con el propósito de atender las necesidades más urgentes de los países miembros de la OEA, especialmente los de menor desarrollo relativo. Los proyectos del FEMCIDI se orientan a mejorar la capacidad de los recursos humanos y a fortalecer las instituciones de gobierno, y actúan como detonantes de programas de desarrollo más amplios y de mayor alcance.

## CONCLUSIONES

Los antivenenos ofídicos y aracnídicos son productos *semi-huérfanos*. Esta situación plantea un escenario favorable para fortalecer la producción de antivenenos por los laboratorios públicos. Los gobiernos deberán tomar una decisión política al respecto en aras de la equidad en la salud de la población. A escala de la Región, estas acciones podrían enmarcarse en un programa liderado por la OPS con el cual se mejore la disponibilidad de estos biológicos en centros asistenciales estratégicamente localizados.

Se han identificado doce establecimientos públicos productores de antivenenos en la Región de los cuales los de Brasil y México son los mayores productores públicos. La gestión de estos laboratorios debe ser la propia de una organización industrial productora de bienes tangibles que no soslaye la planificación estratégica. Las autoridades reguladoras nacionales han de ayudar a los laboratorios públicos productores de estos productos biológicos prestándoles asesoramiento y consultoría, pero sin perder la imparcialidad ni el rigor necesarios en la evaluación de sus sistemas de gestión de la calidad.

Las nuevas tecnologías que mejoran la producción a partir de plasma hiperinmune de mamíferos aún están en fase experimental. No se ha encontrado en la bibliografía información sobre su incorporación en las líneas de producción.

### Agradecimiento.

Los autores agradecen a la Profesora y Doctora Mirtha Bisoglio de Jiménez Bonino la lectura crítica del manuscrito original de este artículo.

### Financiación.

Este estudio no ha recibido financiación.
